# Ankylostomiasis

**Published:** 1903-01-17

**Authors:** 


### ANKYLOSTOMIASIS.

In view of the recently reported presence of ankylostomiasis among Cornish miners it is of interest to note some experiments made by Dr. Looss which go to prove the correctness of his former statement as to ankylostoma larvæ entering the human body by the skin as well as by the alimentary canal. A man who offered himself for experiment had his forearm gently smeared with a mixture of charcoal and fæces in which ankylostoma larvæ had been bred. On the 71st day after this ankylostoma eggs were found in his fæces, and since then have been seen several times. Similar experiments were performed on two puppies, both of which quickly succumbed, and were found to have numerous young ankylostoma worms in the jejunum. Care was taken in all the cases to examine the fæces regularly for some weeks prior to the experiments in order to ascertain that the subjects were not already harbouring the worms. The matter is of much interest since on the hypothesis that the infection enters only by the mouth it might be thought that by careful cleansing of the hands before eating the miners might protect themselves from the disease. Dr. Looss's discovery shows, however, that there is danger whenever the skin is exposed to contamination with infected fæces, for at the temperature of the mine the ova rapidly develop into larvæ, and these are capable of passing through the skin, chiefly by way of the hair follicles.

